# Neck/shoulder pain and low back pain among school teachers in China, prevalence and risk factors

**DOI:** 10.1186/1471-2458-12-789

**Published:** 2012-09-14

**Authors:** Pengying Yue, Fengying Liu, Liping Li

**Affiliations:** 1 Injury Prevention Research Center, Medical College of Shantou University, 22 XinLing Road, Shantou, 515041, China

**Keywords:** Risk factors, Neck and shoulder pain, Low back pain, Teachers

## Abstract

**Background:**

School teachers represent an occupational group among which there appears to be a high prevalence of neck and/or shoulder pain (NSP) and low back pain (LBP). Epidemiological data on NSP and LBP in Chinese teachers are limited. The aim of this study was to investigate the prevalence of and risk factors for NSP and LBP among primary, secondary and high school teachers.

**Methods:**

In a cross-sectional study of teachers from 7 schools, information on participant demographics, work characteristics, occupational factors and musculoskeletal symptoms and pain were collected.

**Results:**

Among 893 teachers, the prevalence of NSP and LBP was 48.7% and 45.6% respectively. There was significant association between the level and prevalence of NSP and LBP among teachers in different schools. The prevalence of NSP among female teachers was much higher than that for males. Self-reported NSP was associated with physical exercise (OR 0.55, 95% CI 0.35 to 0.86), prolonged standing (1.74, 1.03 to 2.95), sitting (1.76, 1.23 to 2.52) and static posture (2.25, 1.56 to 3.24), and uncomfortable back support (1.77, 1.23 to 2.55). LBP was more consistently associated with twisting posture (1.93, 1.30 to 2.87), uncomfortable back support (1.62, 1.13 to 2.32) and prolonged sitting (1.42, 1.00 to 2.02) and static posture (1.60, 1.11 to 2.31).

**Conclusions:**

NSP and LBP are common among teachers. There were strong associations with different individual, ergonomic, and occupational factors.

## Background

Attention has been focused on the prevention of occupational injury and disease to promote health among workers. The National Occupational Research Agenda (NORA) in USA states that work-related musculoskeletal disorders (MSD) account for a major component of the cost of work-related illnesses in the United States. Recent estimates of the cost associated with MSD range from $13 to $54 billion annually [1]. MSD represents one of the most common and most expensive occupational health problems in both developed and developing countries. School teachers represent an occupational group among which there appears to be a high prevalence of MSD [2] . The MSD is one of the leading causes for ill health retirement among school teachers [3]. Studies confirm that low back pain is a common problem in both heavy and light manual workers [4-11]. Musculoskeletal complaints, especially of the lower back, neck and shoulders are also common among teachers. Recently, Hong Kong teachers showed a higher prevalence for neck (68.9%), shoulder (73.4%) and low back pain (59.2%) in the past 30 days. It is worth noting that the sample of Hong Kong teachers showed significantly higher prevalence in all single musculoskeletal complaints than the Norway sample in one study [12]. Epidemiological studies have demonstrated that factors such as gender, age, length of employment and awkward posture are associated with higher MSD prevalence rates among teachers [2]. Sunisa and Pornnapa pointed out that among workers including teachers prolonged posture, static works and repetition are the cause of repetitive strain injuries (RSIs), which is one type of MSDs that directly affect the area of upper limb, neck, shoulder and low back [13].

In China, educational issues such as working stress, teacher unemployment, education reforms, language proficiency assessment for teachers, and reduction in the size and number of classes and schools have already drawn much public attention. However, very little attention has been given to somatic health problems of teachers. Therefore, there is a need to study the problem of musculoskeletal pain among school teachers. The aim of this study was to measure the prevalence of NSP and LBP among Chinese teachers and to investigate the role of risk factors.

## Methods

### Location and background of respondents

Between December 11, 2009 and March 15, 2010, seven schools were selected at random from 60 major public schools in Puning, a city in eastern Guang dong Province. Written consent was obtained from the Ministry of Education in the districts where the schools were located. This research was also approved by the Ethics Committee of the Medical College of Shantou University and the Ministry of Education of Puning. Each teacher in the chosen schools was given a consent letter and a document describing the procedure of the study and its objectives. At the weekly school teachers’ meeting, a questionnaire was administered and it took about 20 min to complete.

### Questionnaire

The demographic characteristics in the questionnaire included gender, age, height and weight. Body mass index (BMI) was calculated by the formula weight (kg)/height (meter)^2^. Normal weight was defined as a BMI of ≤23.9 kg/m^2^, overweight as BMI 24.0-27.9 kg/ m^2^ and obesity as BMI ≥28.0 kg/m^2^[14]. Also, lifestyle (smoking, hours of housework and physical exercise per week) were surveyed. The work characteristics were assessed using questions for school level, years in current job, work hours per week, how many lessons per week and hours of working with computer per day. The questions used to assess occupational risk factors were derived from the standardized Dutch Musculoskeletal Questionnaire (DMQ) [15]. Occupational factors were measured using a dichotomous scale (No/Yes) during an average working day. Work was categorized as tasks with prolonged standing (≥2 hours per day), sitting (≥4 hours per day), static posture, working with hands above shoulder (≥2 hours per day), lifting of weight with hand ≥20kg, posture characterized by twisting posture (≥2 hours per day), uncomfortable back support, have enough rest time. The questions on musculoskeletal symptoms were assessed according to the Nordic Musculoskeletal Questionnaire (NMQ) and were accompanied by anatomical diagrams depicting the specified sites [16]. The participants were asked whether they had experienced pain in the neck or/and shoulder and low back lasting for more than 1 day during the previous 12 months.

### Statistical analysis

The results were reported as descriptive statistics. Odds ratios (OR) and 95% confidence intervals (95%CI) were calculated to examine the association of NSP and LBP with demographic, individual and occupational risk factors using binary logistic regression. Initially, univariate analyses were calculated, with each of the potential explanatory variables as independent variables and NSP or LBP as the dependent variable. OR were obtained for each potential factor after adjustment for age (as a continuous variable with a unit of analysis of 1 year), and gender. In further multivariate logistic regression, non-significant variables (*P* > 0.05) were excluded, with the exception of age and gender, which remained in the model regardless of statistical significance. Multivariate logistic regression analyses were performed using all retained variables. The final model included terms with a *P* value less than 0.05. All analyses were performed using SPSS version 19.0.

## Results

There were 1050 respondents completed the questionnaire. Questionnaires with more than 90.0% of items completed (n = 893) were included in the analysis. The response rate was 85.0% among the participants. 280 respondents (31.4%) were from primary schools, 264 (29.6%) junior middle schools and 349 (39.0%) secondary schools. The participants had a higher proportion of females (67.0% vs 33.0%) than male. Most respondents had 1-9 years of teaching experience (51.2%), taught in secondary schools (66.3%) and high schools (66.5%). The high school teachers reporting workload was higher than that of secondary and primary school teachers. Details of the demographic and work characteristics are shown in Table 1.

**Table 1:** Demographic, life style and work characteristics of primary (n = 280), secondary (n = 264) and high school teachers (n = 349)

**Characteristics**	**Primary school teachers**	**Secondary school teachers**	**High school teachers**	**Overall**
	**n (%)**	**n (%)**	**n (%)**	**n (%)**
Gender				
Male	28 (10.0%)	88 (33.3%)	179 (51.3%)	295 (33.0%)
Female	252 (90.0%)	176 (66.7%)	170 (48.7%)	598 (67.0%)
Age (yrs)				
19-29	64 (22.9%)	144 (54.5%)	190 (54.4%)	398 (44.6%)
30-39	135 (48.2%)	92 (34.8%)	113 (32.4%)	340 (38.1%)
40-49	65 (23.2%)	26 (9.9%)	36 (10.3%)	127 (14.2%)
≥50	16 (5.7%)	2 (0.8%)	10 (2.9%)	28 (3.1%)
Years in current job (yrs)				
1-9	50 (17.9%)	175 (66.3%)	232 (66.5%)	457 (51.2%)
10-19	146 (52.1%)	70 (26.5%)	77 (22.1%)	293 (32.8%)
20-29	60 (21.4%)	16 (6.1%)	32 (9.2%)	108 (12.1%)
≥30	24 (8.6%)	3 (11.0%)	8 (2.3%)	35 (3.9%)
Work hours per week (h)				
≤40	206 (75.2%)	177 (67.8%)	192 (55.7%)	575 (65.3%)
>40	68 (24.8%)	84 (32.2%)	153 (44.3%)	305 (34.7%)
Hours of lessons per week (h)				
<14	194 (70.3%)	211 (80.5%)	169 (50.0%)	574 (65.5%)
≥14	82 (29.7%)	51 (19.5%)	169 (50.0%)	302 (34.5%)
Hours of working with computer per day (h)				
<4	200 (96.2%)	239 (93.7%)	217 (64.8%)	656 (82.2%)
≥4	8 (3.8%)	16 (6.3%)	118 (35.2%)	142 (17.8%)
Hours of physical exercise per week (h)				
<7	196 (82.0%)	184 (70.5%)	278 (86.9%)	658 (80.2%)
≥7	43 (18.0%)	77 (29.5%)	42 (13.1%)	162 (19.8%)
Hours of doing housework per week				
<20	185 (68.5%)	210 (80.5%)	301 (92.3%)	696 (81.2%)
≥20	85 (31.5%)	51 (19.5%)	25 (7.7%)	142 (18.8%)

Detailed descriptive statistics for demographic and labor characteristics of female and male teachers are shown in Table 2. Age distribution was similar (32.18 ± 0.31 vs 32.25 ± 0.46). The smoking ratio for men was higher than that for women, 35.0% and 7.0%, respectively. A higher proportion of men (42.3%) reported work time per week >40 h compared with women (30.9%), and a higher proportion of women (25.7%) reported housework time per week ≥20 h compared with men (4.6%).

**Table 2:** Descriptive statistics of individual, life style and work characteristics among female and male teachers

**Characteristics**	**Women (n = 598)**	**Men (n = 295)**	**Total (n = 893)**	***P*value**
Age (yrs)	32.18 ± 0.31	32.25 ± 0.46	32.21 ± 10.61	0.90
Seniority (yrs)	11.13 ± 8.23	9.56 ± 8.72	10.61 ± 8.42	0.01
Stature (cm)	158.67 ± 4.06	170.2 ± 4.98	162.47 ± 6.98	**<0.001**
Weight (kg)	52.35 ± 6.93	65.43 ± 9.02	56.67 ± 9.84	**<0.001**
Body mass index (kg/m^2^)	20.82 ± 2.62	22.54 ± 2.77	21.39 ± 2.79	**<0.001**
Smoking				**<0.001**
Never or seldom smoked	564 (99.3%)	186 (65.0%)	750 (87.8%)	
Current or past smoker	4 (7.0%)	100 (35.0%)	104 (12.2%)	
Work hours per week (h) >40 h	182 (30.9%)	123 (42.3%)	305 (34.7%)	**0.001**
Housework per week (h) ≥20 h	148 (25.7%)	13 (4.6%)	161 (18.8%)	**<0.001**
Physical exercise per week (h) ≥7 h	96 (17.7%)	66 (23.7%)	162 (19.8%)	**0.04**

*P* Values were derived from either Student's *t*-test for quantitative data or the *χ*2 test for categorical data.

Statistically significant differences (*P* < 0.05) are marked in bold.

The one-year prevalence of NSP and LBP were found to be 48.7% and 45.6% respectively. Results demonstrated that females had a significantly higher prevalence of NSP (51.7% vs 42.7%, *P* = 0.01) (Table 3). Moreover, results showed that the age group with the highest prevalence of NSP and LBP was 40–49, there was significant difference among different age groups in the prevalence of LBP (*P* = 0.03). Senior middle school teachers had the highest prevalence of NSP and LBP. Similarly, there were significant differences among different school levels in the prevalence of NSP and LBP (*P* < 0.001).

**Table 3:** One year prevalence of NSP and LBP in relation to individual factors

**Variable**	**%with NSP (no. of teachers with NSP/Total number)**	***P *value**	**%with LBP (no. of teachers with LBP/Total number)**	***P *value**
Gender		**0.01**		0.1
Male	42.7 (126/295)		41.7 (123/295)	
Female	51.7 (309/598)		47.5 (284/598)	
Age (yrs)		0.18		**0.03**
19-29	46.2 (184/398)		41.2 (164/398)	
30-39	48.5 (165/340)		46.8 (159/340)	
40-49	57.5 (73/127)		55.9 (71/127)	
≥50	46.4 (13/28)		46.4 (13/28)	
Seniority (yrs)		0.25		0.16
1-9	46.0 (210/457)		42.7 (195/457)	
10-19	51.5 (151/293)		47.4 (139/293)	
20-29	49.1 (53/108)		48.1 (52/108)	
≥30	60.0 (21/35)		60.0 (21/35)	
Body mass index (kg/m2)		0.44		0.08
≤23.9	49.4 (344/697)		44.5 (310/697)	
24-27.9	43.5 (57/131)		47.3 (62/131)	
≥28	53.5 (8/15)		73.3 (11/15)	
School level		**<0.001**		**<0.001**
Primary school	48.9 (137/280)		46.8 (131/280)	
Junior middle school	31.4 (83/264)		28.0 (74/264)	
Senior middle school	61.6 (215/349)		57.9 (202/349)	

*χ*2 test for categorical variables. Statistically significant differences (*P* < 0.05) are marked in bold.

Table 4 shows that female gender was significantly associated with NSP, but not with LBP, and physical exercise were significantly associated with NSP and LBP. LBP was closely associated with BMI of those who were obese.

**Table 4:** Association between demographic and life style factors and reporting of NSP and LBP in the past 12 months

**Factors**	**NSP**	**LBP**
	**OR (95% CI)**	**OR (95% CI)**
Gender		
Male	1	1
Female	1.44 (1.10-1.90)*	1.27 (0.96-1.68)
Age (yrs)		
19-29	1	1
30-39	1.00 (0.59-1.64)	1.02 (0.61-1.70)
40-49	1.25 (0.45-3.52)	1.14 (0.41-3.20)
≥50	0.77 (0.14-4.15)	0.63 (0.12-3.41)
Body mass index (kg/m2)		
≤23.9	1	1
2 4-27.9	0.83 (0.56-1.24)	1.12 (0.75-1.67)
≥28	1.18 (0.42-3.34)	3.35 (1.05-10.72)*
Smoking		
Never or seldom smoked	1	1
Current or past smoker	1.00 (0.61-1.59)	0.93 (0.58-1.51)
Hours of physical exercise per week (h)		
<7	1	1
≥7	0.46 (0.32-0.67)*	0.54 (0.38-0.78)*
Hours of doing housework per week (h)		
<20	1	1
≥20	1.39 (1.00-2.00)	1.16 (0.81-1.66)

All OR are adjusted for age as a continuous variable and gender.

*Wald test, *p* < 0.05.

Table 5 shows that years in current job and number of hours worked per week were not associated with both NSP and LBP. The hours of working with computer ≥4 h/day was associated with NSP but not with LBP. Hours of lessons ≥14 h/week, prolonged standing, sitting, static posture and holding the neck in a forward bent posture, were all associated with NSP and LBP. Have enough rest time was also associated with LBP.

**Table 5:** Association between work characteristics and occupational factors and reporting of NSP and LBP in the past 12 months

**Factors**	**NSP**	**LBP**
	**OR (95% CI)**	**OR (95% CI)**
Years in current job (yrs)		
1-9	1	1
10-19	1.02 (0.66-1.59)	0.81 (0.52-1.27)
20-29	0.79 (0.35-1.80)	0.56 (0.25-1.27)
≥30	1.17 (0.33-4.11)	0.66 (0.19-2.32)
School level		
Primary school	1	1
Secondary school	0.61 (0.42-0.89)*	0.57 (0.39-0.83)*
High school	2.48 (1.71-3.59)*	2.22 (1.54-3.20)*
Work hours per week (h)		
≤40	1	1
>40	1.26 (0.95-1.67)	0.96 (0.72-1.27)
Hours of lessons per week (h)		
<14	1	1
≥14	1.95 (1.46-2.60)*	1.73 (1.31-2.30)*
Hours of working with computer per day (h)		
<4	1	1
≥4	1.82 (1.25-2.66)*	1.13 (0.91-1.91)
Prolonged standing		
No	1	1
Yes	2.23 (1.48-3.78)*	1.88 (1.25-2.84)*
Prolonged sitting		
No	1	1
Yes	1.78 (1.36-2.34)*	1.60 (1.22-2.10)*
Prolonged static posture		
No	1	1
Yes	3.20 (2.42-4.24)*	2.58 (1.96-3.42)*
Working with hands above shoulder		
No	1	1
Yes	1.55 (1.18-2.03)*	1.62 (1.23-2.13)*
Lifting of weight with hand ≥20 kg		
No	1	1
Yes	1.07 (0.66-1.74)	1.40 (0.86-2.27)
Holding the neck in a forward bent posture for a long time		
No	1	1
Yes	2.18 (1.66-2.87)*	2.33 (1.77-3.07)*
Have enough rest time		
No	1	1
Yes	0.82 (0.63-1.08)	0.68 (0.52-0.90)*
Posture characterized by twisting		
No	1	1
Yes	1.94 (1.41-2.69)*	2.53 (1.83-3.52)*
Uncomfortable back support		
No	1	1
Yes	2.60 (1.98-3.46)*	2.64 (2.00-3.50)*

All OR are adjusted for age as a continuous variable and gender.

*Wald test, *p* < 0.05.

In the final multivariate model (Table 6), for individual factors, gender and physical exercise remained in the last model. The NSP and LBP were positively associated with high school teacher. Secondary school level remained associated with decreased odds of reporting NSP and LBP as compared to primary school teachers. Occupational factors of prolonged standing, sitting and static posture, uncomfortable back support and twisting posture remained associated with NSP and LBP in the final model.

**Table 6:** Multivariate model for association between NSP and LBP in the past 12 months

	**NSP**	**LBP**
**Factors**	**OR (95% CI)**	**OR (95% CI)**
Female gender	1.84 (1.25-2.71)*	1.41 (0.97-2.07)
Physical exercise per week (h) ≥7 h	0.55 (0.35-0.86)*	0.71 (0.46-1.09)
School level		
Primary school	1	1
Secondary school	0.96 (0.60-1.54)	0.68 (0.42-1.08)
High school	2.35 (1.43-3.85)*	2.01 (1.24-3.27)*
Hours of lessons per week (h) ≥14 h	1.21 (0.83-1.76)	1.05 (0.72-1.52)
Hours of working with computer per day (h) ≥4 h	1.02 (0.63-1.65)	0.71 (0.44-1.14)
Prolonged standing	1.74 (1.03-2.95)*	1.48 (0.88-2.50)
Prolonged sitting	1.76 (1.23-2.52)*	1.42 (1.01-2.02)*
Prolonged static posture	2.25 (1.56-3.24)*	1.60 (1.11-2.31)*
Working with hands above shoulder	1.21 (0.86-1.71)	1.27 (0.90-1.79)
Holding the neck in a forward bent posture for a long time	1.12 (0.77-1.63)	1.32 (0.91-1.91)
Have enough rest time	1.42 (0.98-2.04)	1.08 (0.76-1.55)
Posture characterized by twisting	1.16 (0.77-1.73)	1.93 (1.30-2.87)*
Uncomfortable back support	1.77 (1.32-2.55)*	1.62 (1.13-2.32)*

All OR are mutually adjusted and adjusted for age as a continuous variable and gender.

*Wald test, *p* < 0.05.

## Discussion

The results of this study show that NSP and LBP are common in school teachers in Puning, China.

### The prevalence of NSP and LBP

The first aim of this study was to estimate the 12-months prevalence of NSP and LBP among school teachers in Puning. Our study identified 48.7% and 45.6% prevalence of NSP and LBP among teachers. Parallels can be drawn to other studies where 42.5-47.9% and 43.8-74.9% of Turkish school teachers reported having experienced neck pain and low back pain respectively, while 28.7-55.9% had experienced MSD symptoms in the shoulder area [17,18]. In Brazil and Malaysia, 41.1% and 40.4% of elementary school teachers reported low back pain [19,20]. Another study of Brazil obtained data showed the presence of pain in the trapezius muscle region, on the left side, in 52.5%; and, on the right side, in 50.6% among elementary school teachers [21]. Other studies where 40.0% of Chinese primary school teachers and 34.8% of French school teachers also reported back pain [7,22]. In Chinese Hong Kong, studies of secondary school teachers reported a 12-month prevalence of neck pain at 64.4% and 66.7% [23,24]. The disorders seem to be most common in the neck, shoulders and low back among teachers. Our results are generally consistent with these prior studies in school teachers. It is important to pinpoint hazards for developing prevention strategies.

### Individual factors

Many individual factors including age, gender and BMI may play a role in causing NSP and LBP. Our results showed that gender and physical exercise were related to NSP. The prevalence of NSP was substantially higher among women (51.7%) than men (42.7%) in our study, which is consistent with previous studies [25,26]. Women appear to consistently report more neck, shoulder and upper extremity symptoms than men [12,18,23,24,27,28]. In our study, even though males had a higher BMI, longer employment than females, a significant higher proportion of smokers and often worked time >40 h, females were significantly at risk for NSP. The gender difference may be explained by many factors, one of which could be that the women had in which they were more likely suffered emotional exhaustion compared with men among teachers [29]. This may also explained partially by women having a lower pain threshold than males. Torgen et al. suggested that pressure pain thresholds increased with muscle strength and Chiu et al. found that the isometric neck muscle strength in all directions for men was 1.2–1.7 times those in women [30,31]. Moreover, we found females bore more heavy housework than male in daily life, and some authors suggest that differentials in household task participation may explain WMSDs differences between men and women [25,32].

### Work-related physical and occupational factors

Teachers at the senior middle school level reported the highest prevalence of NSP and LBP, in comparison to those at the primary and secondary school levels, which is consistent with previous studies [33]. The teachers who worked in high schools suffered significantly higher risks of NSP and LBP. One of the reasons could be that senior middle school teachers have to deal with more examinations and are under higher pressure to graduate students. So they experience more psychological stress and a higher work load than others. In the present study, teachers who worked in senior middle schools also had the highest work load in comparison to those who worked in other levels of schools. Emotional exhaustion correlates with the high numbers of weekly lessons [34]. Work activities that involve heavy lifting, awkward postures, bending, twisting or stooping, prolonged sitting or standing and repetitive motions may contribute to the development of MSD [35-37]. Activities of sustained sitting of frequent reading, marking of assignment and in front of computer, standing up teaching in class, repetitively overhead writing on board are also unsafe act and favorable to the development of NSP, LBP and upper limb pain which found in teachers [20,23,38]. Studies have also confirmed that sitting for more than 3 hours daily could be a risk factor for LBP [39,40]. But Lis and colleagues, in their systematic review, found that sitting itself does not increase the risk of LBP, but sitting for more than half a workday, combined with whole-body vibration and/or awkward postures, does increase the likelihood of having LBP, and it is the combination of those risk factors that leads to the greatest increase in LBP [41]. Epidemiological studies show a significant association among uncomfortable back support and LBP [42,43]. Moreover, our study further confirmed these findings in school teachers. In the present study prolonged sitting and static posture and uncomfortable back support were positively associated with NSP and LBP. In addition, prolonged standing was closely associated with NSP alone.

Our data demonstrates significant school levels differences in the experience of NSP and LBP. Moreover, our data point to senior middle school teachers in which there is a very great need to study workload stress factors and devise adequate preventive and interventional action.

Our study had several limitations. Information about musculoskeletal symptoms and related factors were obtained by the self-reporting method and the nature of this retrospective questionnaire survey, it is difficult to rule out the possibility of recall bias, which may lead to over-or underestimation. Furthermore, as a cross sectional study, only associations can be established but no inferences of causality can be made.

## Conclusion

The prevalence of NSP and LBP among teachers in Puning, a developing city of China, is high and comparable to prevalence in other countries. Different individual, ergonomic, and occupational factors were important associations of NSP and LBP. Hence effective preventive strategies need to address this area. Further, studies on different interventional models are required to develop an effective preventive strategy for these relatively common and underestimated problems.

## Abbreviations

NSP: Neck and/or shoulder pain; LBP: Low back pain; WMSDs: Work-related Musculoskeletal disorder; NMQ: Nordic Musculoskeletal Questionnaire; DMQ: Dutch Musculoskeletal Questionnaire; NORA: National Occupational Research Agenda.

## Competing interests

The authors declare that they have no competing interests.

## Authors’ contributions

LF made substantial contribution to conception and study design. LF was involved in data collection. YP was involved in statistical analysis and drafting the manuscript. LL critically revised the manuscript. All authors read and approved the final manuscript.

## Pre-publication history

The pre-publication history for this paper can be accessed here:

http://www.biomedcentral.com/1471-2458/12/789/prepub
